# Assessing bed net use and non-use after long-lasting insecticidal net distribution: a simple framework to guide programmatic strategies

**DOI:** 10.1186/1475-2875-9-133

**Published:** 2010-05-18

**Authors:** Jodi L Vanden Eng, Julie Thwing, Adam Wolkon, Manisha A Kulkarni, Ayub Manya, Marcy Erskine, Allen Hightower, Laurence Slutsker

**Affiliations:** 1 Centers for Disease Control and Prevention, Center for Global Health, Division of Parasitic Diseases and Malaria, 4770 Buford Hwy MS F-22, Atlanta, GA 30341, USA; 2 HealthBridge, 1105-1 Nicholas Street, Ottawa, ON, K1N 7B7, Canada; 3 Division of Malaria Control, Ministry of Health, Kenya, PO Box 19982-00202, Nairobi; 4 International Federation of Red Cross and Red Crescent Societies, 17, ch. des Crêts/Petit - Saconnex, PO Box 372, 1211 Geneva 19, Switzerland

## Abstract

**Background:**

Insecticide-treated nets (ITNs) are becoming increasingly available to vulnerable populations at risk for malaria. Their appropriate and consistent use is essential to preventing malaria, but ITN use often lags behind ITN ownership. In order to increase ITN use, it is necessary to devise strategies that accurately identify, differentiate, and target the reasons and types of non-use.

**Methods:**

A simple method based on the end-user as the denominator was employed to classify each individual into one of four ITN use categories: 1) living in households not owning an ITN; 2) living in households owning, but not hanging an ITN; 3) living in households owning and hanging an ITN, but who are not sleeping under one; and 4) sleeping under an ITN. This framework was applied to survey data designed to evaluate long-lasting insecticidal nets (LLINs) distributions following integrated campaigns in five countries: Togo, Sierra Leone, Madagascar, Kenya and Niger.

**Results:**

The percentage of children <5 years of age sleeping under an ITN ranged from 51.5% in Kenya to 81.1% in Madagascar. Among the three categories of non-use, children living in households without an ITN make up largest group (range: 9.4%-30.0%), despite the efforts of the integrated child health campaigns. The percentage of children who live in households that own but do not hang an ITN ranged from 5.1% to 16.1%. The percentage of children living in households where an ITN was suspended, but who were not sleeping under it ranged from 4.3% to 16.4%. Use by all household members in Sierra Leone (39.9%) and Madagascar (60.4%) indicate that integrated campaigns reach beyond their desired target populations.

**Conclusions:**

The framework outlined in this paper provides a helpful tool to examine the deficiencies in ITN use. Monitoring and evaluation strategies designed to assess ITN ownership and use can easily incorporate this approach using existing data collection instruments that measure the standard indicators.

## Background

The efficacy and cost-effectiveness of insecticide-treated nets (ITNs) in reducing malaria related morbidity and mortality is well-known [1,2] and in recent years has led to massive efforts to distribute millions of free or highly subsidized ITNs to vulnerable populations in sub-Saharan Africa [3-6]. In 2005, the Roll Back Malaria Partnership (RBM) set the goal for 2010 to achieve 80% coverage of children <5 years of age and pregnant women with protective measures such as ITNs [7]. More recently, the Global Malaria Action Plan called for rapid scale-up to universal population coverage for all people at risk for malaria [8]. This scale-up in ITN delivery necessitates an equivalent increase in monitoring and evaluation (M&E) efforts in order to determine the impact of ITN distributions as well as prioritize future programmes.

Typical M&E strategies report on indicators agreed upon and formalized by the RBM Monitoring and Evaluation Reference Group (MERG). Two important core ITN indicators for malaria control programmes are the proportion of households owning an ITN and the proportion of vulnerable populations sleeping under an ITN. Using these measures, many studies have shown that efforts to increase ITN ownership have made tremendous progress; however, most sub-Saharan countries remain well below RBM targets for ownership [9]. In addition, these evaluations have consistently found ITN use (vulnerable groups sleeping under an ITN) lower than household ownership [10-12]. In order to reach the RBM goals for bed net coverage and increase ITN use, it will be useful for programmes to identify and address the determinants of ITN use and non-use.

The two core RBM bed net indicators provide good measures of ITN ownership and use, but in and of themselves cannot provide measures for non-use. The difference between household ITN ownership and individual use cannot be used to estimate non-use because these indicators have different denominators (households and persons, respectively). As a result, there exists literature investigating determinants of bed net use and non-use with a variety of different methods. One method is to restrict analyses to households that already own an ITN in order to observe factors of use other than access [12,13]. For example, in households owning ITNs, children < 5 and women of reproductive age are more likely to sleep under ITNs than other household members, perhaps confirming the effectiveness of messaging that often accompanies distribution or marketing campaigns targeting these high risk groups [14,15]. Data on persons who did not use an ITN the previous night may prove equally informative. A bed net study in Kenya looked at reasons given for not hanging a net and factors that increase or decrease the likelihood that a net will be used; the most important reason for non-adherence was disruption of sleeping arrangements [16].

Following mass distribution campaigns some malaria control programmes implement intervention strategies aimed at increasing ITN coverage. Information, education, and communication/behaviour change communication (IEC/BCC) interventions primarily target and encourage people to hang and sleep under existing bed nets. Hang-up campaigns, send volunteers to selected homes and teach the occupants the importance of sleeping under an ITN, demonstrate how to hang a net, and hang one or more of the household's nets. Keep-up distributions, social marketing, subsidized nets, and distribution through other public health services such as antenatal care clinics aim to improve access to ITNs. These strategies for increasing ITN coverage can be loosely classified into three groups: 1) those aimed at improving ITN access, 2) those encouraging hanging ITNs, and 3) those targeting individuals to encourage sleeping under an existing ITN. Data obtained from surveys evaluating the core RBM bednet indicators can be used to determine what proportion of individuals currently not sleeping under an ITN would be targeted by each of these three categories. Results identifying specific areas of non-use will enable malaria programmes to make informed decisions about which intervention strategies may be more cost-effective to improve ITN coverage.

Although population based indicators of ITN non-use could indeed be programmatically useful, none currently exist. This paper presents a simple framework that supplements information provided by the two core RBM bed net indicators through applying an easy-to-use method to evaluate the different categories of non-use. This technique can be easily applied to data commonly collected on national household cluster survey instruments such as Malaria Indicator Surveys (MIS) and Demographic and Health Surveys (DHS). To demonstrate, this approach was applied to data from household cluster surveys designed to evaluate long-lasting insecticidal net (LLIN) coverage following integrated child health campaigns in five countries.

## Methods

### Integrated campaigns

National integrated child health campaigns distributed LLINs and other child health commodities between 2004 and 2006 in five countries: Togo, Niger, Kenya, Madagascar, and Sierra Leone (Table 1). The campaigns were nationwide in Togo, Niger, and Sierra Leone, and in a sub-national area of malaria risk in Kenya and Madagascar. With the exception of Kenya, where ITNs had previously been distributed through a subsidized voucher system in antenatal and vaccination clinics and to a limited degree through the private sector, very few ITNs were present in the other countries prior to these campaigns. In Niger and Kenya, large numbers of conventional nets, usually untreated, were present. Following mass LLIN distribution, hang-up campaigns were implemented in selected areas in each country.

**Table 1:** Summary of campaigns and surveys in each country

Country	Number of LLINs Distributed and Strategy	Population	Dates of Campaign	Dates of Survey	Number of Households Surveyed	Number of Children (People)* Surveyed
Togo	850,000; one per child 9-59 months	5,548,702	Dec 2004	Sept 12-Oct 7, 2005	3523	4134

Niger	2,100,000; one per mother with ≥ 1 child < 5 years of age	12,525,094	Dec 2005, March 2006	Sept 11-Oct 2, 2006	2450	3113

Kenya	3,400,000; one net per child <5 years of age	34,707,817	July 2006, Sept 2006	Oct 18-Nov 1, 2006	2059	1757

Madagascar	1,500,000; one per child <5 years of age, max 2 per household	20,653,556	Oct 2007	April 11-May 1, 2008	4320	3355 (18718)

Sierra Leone	875,000; one per child <5 years of age, max 2 per household	6,294,774	Nov 2006	Oct 29-Nov 17, 2007	5186	4674 (22149)

*The numbers in parenthesis provide the total number of people interviewed for Madagascar and Sierra Leone, the two countries in which data on all household members was collected.

### Surveys

Post-campaign evaluation surveys were conducted during the rainy season following the integrated campaigns in these five countries (Table 1) [6,17-19]. These surveys all employed sampling designs and questionnaires that incorporated rosters of nets and household occupants for assessing net ownership, hanging and use similar to the MIS. These methods have been described in detail elsewhere [6]. In brief, a community-based, stratified two-stage cluster sample design was used followed by a selection of a simple random sample of households. Data provided by the most recent census that included population estimates at regional, local, and enumeration area (EA) or village level were used as the sampling frame. Probability proportional to size sampling (PPS) was used first to select regions/districts, and again at the second stage of sampling to choose the primary sampling unit (a village or EA). If a village/EA was inaccessible for logistical reasons, a randomly selected alternate was used. Personal digital assistants (PDAs) equipped with global positioning systems (GPS) were used for data collection, as previously described [20]. After GPS-mapping of the selected community, households were selected for interview using simple random sampling implemented on the PDA. The same day of the mapping, the teams returned to the selected households to administer the questionnaire by PDA (developed using Visual CE; Syware Inc., Cambridge, MA, USA). Households were revisited if no one was available for interview on the first attempt; if no one was available after 2 attempts the interviewer continued to the next randomly selected household on the list until the desired number of households was obtained. For each cluster, the number of households in the sampled list was increased in size at the sampling stage to cover potential non-response. Therefore, the probability of inclusion for a household within a cluster was the proportion of households selected times the response rate for that cluster. No alternate was used if a household refused to participate, with the exception of Niger, in which a randomly selected alternate was used. Refusal information was not recorded for Kenya and Togo.

All protocols were reviewed and approved by the Centers for Disease Control and Prevention Human Subjects Research Office, and ethical clearance was obtained from the ethical and human subjects committees in the respective countries. Participants gave verbal informed consent to answer the questionnaire.

### Data analysis

The two core RBM outcome indicators were calculated following standard guidelines [21]. Household ITN possession was defined as the number of households surveyed with at least one ITN over a denominator of the total number of households surveyed. The level of ITN coverage of children was defined as the number of children <5 years of age who slept under an ITN the previous night over a denominator of the total number of children <5 years of age who spent the previous night in surveyed households. Another commonly used indicator, the proportion of ITNs hanging, is also presented here for completeness. This was calculated as the number of ITNs suspended over a sleeping space the previous night, over a denominator of the total number of ITNs in households surveyed. Results for the two RBM core indicators in Madagascar and Niger have been published elsewhere and are repeated here for completeness [6,17].

**Figure 1:**
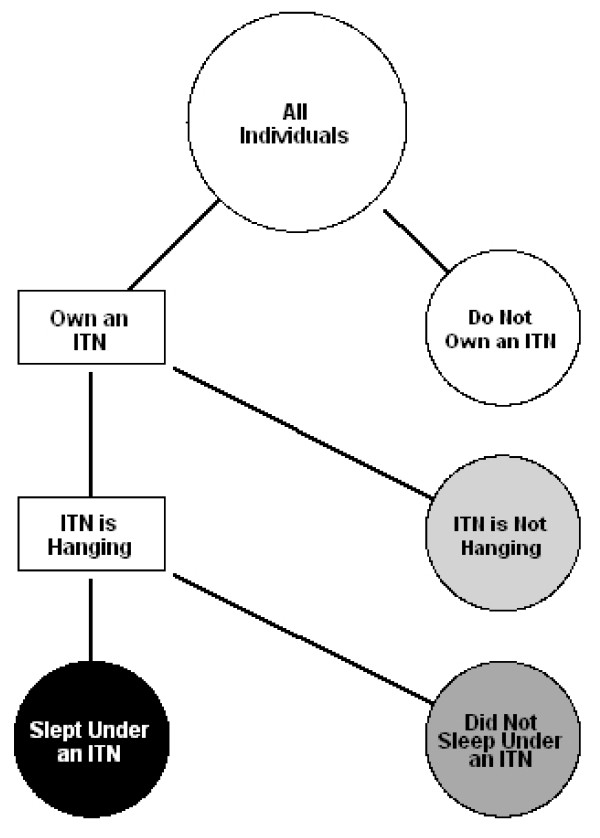
**Flowchart representing the four categories of ITN use**. ITN use categories are represented as circles. White: living in households with no ITN present; light grey: living in households owning but not hanging an ITN; dark grey: living in households that have an ITN hanging but are not sleeping under an ITN; and black: sleeping under an ITN.

Based on survey responses about ownership, bed net characteristics, and individual use, individuals were classified into one of four categories of ITN use: 1) living in households with no ITN present; 2) living in households owning but not hanging an ITN; 3) living in households that have an ITN hanging but who are not sleeping under an ITN; and 4) sleeping under an ITN (Figure 1). These four mutually exclusive categories all use a common denominator: all members of the target group of interest. For example, with data from child health campaigns the common denominator would likely be the number of children < 5 years of age who spent the previous night in surveyed households; and for the first category of ITN use the numerator would be the number of those children who live in a household that does not own an ITN. The first three categories represent non-use and comprise the deficit between the observed ITN use and 100% coverage. The last category represents the core RBM ITN use indicator described above.

Analyses were performed using SAS survey procedures (SurveyFreq and SurveyLogistic, version 9.1, SAS Institute, Cary, NC). Analyses were performed separately for each country and were stratified where appropriate. Estimates were weighted based on the probability of being selected. To account for the cluster sampling design, standard errors of estimates were clustered by household or enumeration area in the analyses.

In general, a household was defined as a male or female head of household and his/her dependents including wives and unmarried children; usually the group of people sharing a cooking pot or common source of income. However, the definition varied somewhat among countries. An ITN was defined as any LLIN or a conventional net treated within the past 12 months [7]. Pregnancy status was based on self-report. An individual who reported having slept under a net the previous night was defined as having used the net. A net was considered hanging if it was reported to be suspended over a sleeping space the previous night.

The data presented are representative of households available and consenting to an interview in areas included in the campaigns and not nationally representative in Kenya and Madagascar. Results are presented for children <5 years of age, the target group for the integrated campaigns. However, when available, this approach could be applied to all members of the household or certain subgroups (e.g. wealth quintile, rural or urban setting, and gender). This information was collected in two of the surveys (Sierra Leone and Madagascar), and results are presented for subgroup analyses for these countries by age groups (infants, children <5 years of age, older children, pregnant women, other adults).

## Results

Response rates were fairly high with 90.8% (Kenya), 86.9% (Niger), 91.7% (Togo), 92.7% (Sierra Leone) and, 92.8% (Madagascar) of the households available for interview.

### Core RBM indicators of household ITN ownership and individual use, and percentage of ITNs hanging

**Figure 2:**
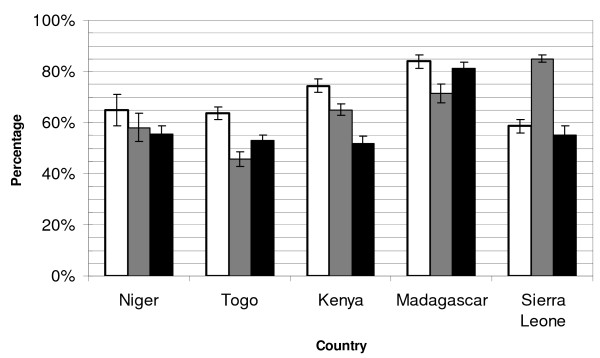
**Indicators of ITN ownership and use: survey results following integrated campaigns targeting children <5 years of age in Niger, Togo, Kenya, Madagascar, and Sierra Leone**. Bars represent: white: percentage of households that own an ITN (denominator: all households); dark: percentage of ITNs hanging (denominator: all ITNs); and black: percentage of all children less than 5 years of age sleeping under an ITN (denominator: all children less than 5 years of age). Note: household ownership and ITN use by children less than 5 years of age are standard RBM outcome indicators.

The percentage of children <5 years of age sleeping under an ITN approached or met RBM targets during the rainy season, ranging from 51.5% (95% CI: 48.5-54.4) in Kenya to 81.1% (95% CI: 78.8-83.5) in Madagascar following integrated child health campaigns (Figure 2). In all five countries, levels of ownership were higher than use by children < 5 years. The percent of households owning an ITN ranged from 58.6% (95% CI: 55.9-61.3) in Sierra Leone to 83.9% (95% CI: 81.3-86.5) in Madagascar.

The percent of ITNs suspended over a sleeping space the previous night varied greatly among the countries. Although Sierra Leone had one of the lowest levels of ownership and use, it had the highest hanging percentage at 85.0% (95% CI: 83.5-86.5). Niger and Kenya had hanging percentages that lie between ownership and usage (58.0%, 95% CI: 52.6-63.5 and 65.1%, 95% CI: 62.8-67.4 respectively), whereas Togo (45.7%, 95% CI: 42.9-48.5) and Madagascar (71.5%, 95% CI: 67.9-75.1) had a smaller percentage of ITNs hanging than either ownership or use.

### Evaluating the three categories of ITN non-use by children <5 years of age

**Figure 3:**
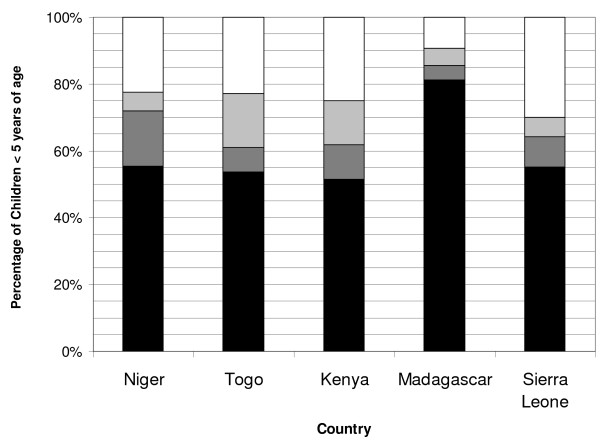
**Percentage of all children <5 years of age in each of the four ITN use categories, in Niger, Togo, Kenya, Madagascar, and Sierra Leone during the rainy season**. Denominator includes all children <5 years of age. ITN use categories are: white: living in households with no ITN present; light grey: living in households owning but not hanging an ITN; dark grey: living in households that have an ITN hanging but are not sleeping under an ITN; and black: sleeping under an ITN.

Children <5 years of age living in households without an ITN make up the largest of the three categories of non-use despite the efforts of the integrated campaign. In four of the five countries, over 20% of all children surveyed lived in households that did not possess an ITN. Madagascar had the lowest proportion at 9.4% (95% CI: 7.6-11.2), whereas in Sierra Leone nearly one-third of the children lived in a house that did not own an ITN (30.0%, 95% CI: 26.1-33.9). In Niger, Madagascar, and Sierra Leone, 5.1-5.9% of all children lived in households that owned but did not hang an ITN; while in Togo and Kenya, this was 16.1% (95% CI: 14.4-17.8) and 13.0% (95% CI: 10.9-15.2), respectively. Children living in households that owned and hung an ITN, but did not sleep under it ranged from 4.3% (95% CI: 3.1-5.6) in Madagascar to 16.4% (95% CI: 14.1-18.7) in Niger (Figure 3).

### Untreated net use by children <5 years of age

In some countries, untreated nets are also present in great numbers in the community. In Niger over two-fifths, (43.8%, 95% CI: 40.5-47.1) and in Kenya over one-fifth (23.2%, 95% CI: 20.4-26.0) of children <5 years of age lived in households that own one or more untreated nets in addition to one or more ITNs. Among children in households with both types of nets hanging, approximately one-third slept under the untreated net instead of the ITN (30.9% Niger, 31.8% Kenya). This accounts for 11.5% (95% CI: 9.5-13.6) and 4.4% (95% CI: 3.3-5.6) of all children < 5 years of age in Niger and Kenya, respectively.

### Core RBM indicator of ITN use by age groups in Madagascar and Sierra Leone

**Figure 4:**
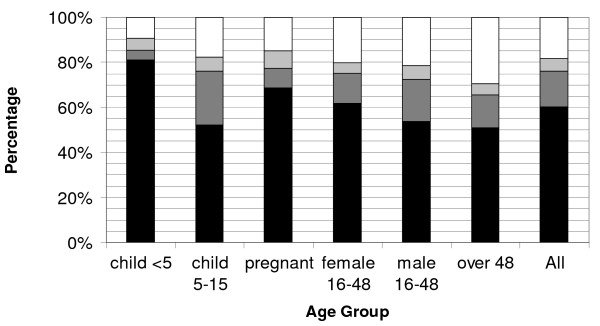
**Age specific breakdown of individuals in each of the four ITN use categories during the rainy season following an integrated campaign targeting children <5 years of age in Madagascar**. Denominator includes all persons in respective age group. ITN use categories are: white: living in households with no ITN present; light grey: living in households owning but not hanging an ITN; dark grey: living in households that have an ITN hanging but are not sleeping under an ITN; and black: sleeping under an ITN. Female 16-48 includes non-pregnant women of reproductive age.

**Figure 5:**
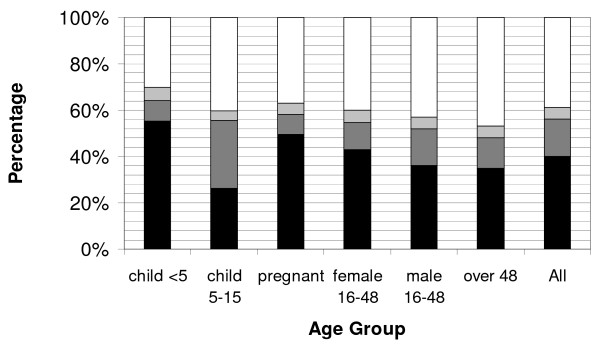
**Age specific breakdown of individuals in each of the four ITN use categories during rainy season following an integrated campaign targeting children <5 years of age in Sierra Leone**. Denominator includes all persons in respective age group. ITN use categories are: white: living in households with no ITN present; light grey: living in households owning but not hanging an ITN; dark grey: living in households that have an ITN hanging but are not sleeping under an ITN; and black: sleeping under an ITN. Female 16-48 includes non-pregnant women of reproductive age.

In two countries, Madagascar and Sierra Leone, data were collected on all household members, not just the vulnerable populations. Although ITN use by children <5 years of age was 81.1% in Madagascar and 55.2% in Sierra Leone, ITN use by persons of all ages was substantially lower, at 60.4% (95% CI: 58.4-62.4) and 39.9% (95% CI: 37.4-42.5), respectively. Variations in ITN use and categories of non-use were observed across age groups in both Madagascar and Sierra Leone (Figures 4 and 5).

The age group with the largest proportion sleeping under an ITN the previous night was children <5 years of age, the target population of the campaign. In contrast, children 5-15 years were one of the least likely groups to sleep under an ITN at 52.0% (95% CI: 48.8-55.2) in Madagascar and 26.2% (95% CI: 23.6-28.8) in Sierra Leone. The proportion of pregnant women sleeping under an ITN varied only slightly from other women of reproductive age: 68.6% (95% CI: 62.6-74.6) vs. 61.7% (95% CI: 59.2-64.2) in Madagascar, and 49.4% (95% CI: 44.0-54.8) vs. 43.1% (95% CI: 40.3-45.8) in Sierra Leone. Men 16-48 years of age and adults > 48 years old had low use, with just over 50% having slept under an ITN the previous night in Madagascar, and roughly 35% in Sierra Leone (Madagascar: 53.8% (95% CI: 51.0-56.6) men 16-48, 51.0% (95% CI: 46.9-55.1) adults > 48 years; Sierra Leone: 36.2% (95% CI: 33.3-39.0) men 16-48, 34.9% (95% CI: 31.3-38.5) adults > 48 years; Figures 4 and 5).

### Evaluating the three categories of ITN non-use by all age groups

Nearly one-fifth of the population living in endemic areas of Madagascar (18.4%, 95% CI: 16.5-20.2) and two-fifths of the population of Sierra Leone (38.9%, 95% CI: 35.8-42.1) are estimated to live in households that did not have an ITN in their possession. In all age groups but one, the greatest category of non-use was living in a household lacking possession of an ITN; ranging from 9.4% (95% CI: 7.6-11.2) and 30.0%(95% CI: 26.1-33.9) of children <5 years of age to 29.6% (95% CI: 25.9-33.3) and 46.9% (95% CI: 42.8-51.5) of adults > 49 years in Madagascar and Sierra Leone respectively. The exception to this was in Madagascar among children 6-15 years of age where the ITN non-use percentage was higher due to not sleeping under a hanging ITN (24.1%, 95% CI: 21.3-26.9) as opposed to not owning an ITN (17.8%, 95% CI: 15.2-20.4).

In both Madagascar and Sierra Leone, roughly 16% of the people lived in households where an ITN was suspended over a sleeping space, but did not themselves sleep under an ITN (15.7%, 95% CI: 14.2-17.1 in Madagascar; 16.1%, 95% CI: 14.5-17.6 in Sierra Leone). Among different age groups, children <5 years of age had the smallest proportion in this category (4.3%, 95% CI: 3.1-5.6 in Madagascar and 8.9%, 95% CI: 7.3-10.5 in Sierra Leone), whereas children 5-15 years old had the largest in both Madagascar (24.1%, 95% CI: 21.3-26.9) and Sierra Leone 29.4% (95% CI: 26.3-32.5) (Figures 4 and 5). The majority of people who lived in households with an ITN hanging but who did not sleep under one were in households that only possessed one ITN (Sierra Leone: 70.7%, Madagascar 60.1%) and approximately 90% lived in households that owned no more than 2 ITNs (Sierra Leone: 92.4%; Madagascar 89.0%).

People living in households that owned but did not hang an ITN made the smallest contribution to non-use in every age group, never reaching 10% (Figures 4 and 5). Overall, this included 5.1% (95% CI: 4.2-5.9) of the total population in Sierra Leone, and 5.6% (95% CI: 4.5-6.7) in Madagascar.

### Reasons for non-use among persons in households with an ITN hanging

During the interview, if a person responded that they did not sleep under a net, a follow up question was asked to determine the reasons why the person was not sleeping under a net. In Madagascar, 75.2% (95% CI: 70.2-80.2) of the persons living in households with ITNs hanging but who did not sleep under an ITN replied either someone else was using the net or there were not enough nets available (breakdown: 27.5% said someone else was using the net, 42.2% said there were not enough nets available, 5.5% gave both reasons). In Sierra Leone, among persons in households with ITNs hanging 33.9% (95% CI: 23.2-44.5) of all people responded the main reason they were not sleeping under a net was 'someone else was sleeping under it' and 45.2% (95% CI: 38.9-51.4) responded 'other' ('not enough nets available' was not offered as a choice). In both countries, when looking across age groups there was little variation in reasons for not sleeping under a net, and among the other possible reasons offered, none exceeded 10% of the respondents.

## Discussion

While many sub-Saharan African countries have made great progress in ITN scale-up, much work remains. An accurate assessment to identify the gaps in household ownership and individual use is a crucial step in devising evidence-based and country-specific strategies to increase population coverage with ITNs and work towards the interruption of malaria transmission. The framework described here, which uses a common denominator (the individual) to evaluate categories of ITN non-use in addition to evaluating the core RBM indicators of household ITN ownership and individual use, provides an additional tool to help accomplish this mission.

In all five countries included in this analysis, the majority of children <5 years of age slept under an ITN the night before the interview. Among children < 5 years not sleeping under an ITN, the largest proportion lived in households that did not own an ITN, despite the efforts of recent child health campaigns. This result, that the largest category of non-use is directly related to household ITN ownership, indicates access is still a barrier to ITN use. This highlights the need to identify distribution strategies targeting previously unreached households to increase ownership of ITNs.

Children <5 years of age living in households hanging an ITN, but not sleeping under one made up a relatively small proportion of non-use for the countries analysed here, suggesting that caretakers recognize the importance of protecting young children with ITNs. In this case, increasing IEC/BCC messages that promote protection of children < 5 with an ITN may not be the most effective method of increasing overall use since they would only be aimed at a small proportion of children not using ITNs. For countries with the smallest proportion of non-use in this category, it may be better to focus efforts on improving one of the other categories of non-use such as increasing ownership or hanging nets. For countries with a large proportion of individuals in this category, further analysis may be needed to determine if there are sufficient numbers of nets per household to cover the population, indicating the need for IEC/BCC to encourage net use, or if there are insufficient nets available, indicating the need for further distribution. In Madagascar and Sierra Leone, additional questions on the determinants of use found the primary reasons for not sleeping under an ITN among persons in households with an ITN hanging, were related to access to an ITN. This suggests further bed net distribution may be more effective than IEC/BCC messages to increase ITN use.

In Niger, Madagascar, and Sierra Leone children <5 years of age living in households owning, but not hanging an ITN, composed a small proportion of the three categories of non-use; suggesting that further hang-up efforts would not lead to major gains in use. In contrast, in Togo and Kenya, a larger proportion of the target population lived in households that owned but did not hang an ITN, indicating a potential gain might be obtained from further a hang-up campaign, other hang-up activities, or increased IEC/BCC programs to encourage net hanging. Although this type of analysis does not address the effectiveness of hang-up activities related to the campaign, it may help identify where to focus efforts and activities beyond those associated with the campaign (by detecting the largest category of non-use) in a cost effective manner.

Countries with a substantial presence of untreated nets (including LLINs that have surpassed their 2-4 year life expectancy) may want to include untreated nets in their evaluation in order to determine their impact on ITN use in households owning both net types. Use of untreated nets may result from differences in behaviour or household dynamics and therefore require interventions that emphasize the advantages of ITNs (or replacing old LLINs) and encourage their use over untreated nets. Alternatively, it could be a result of a shortage of ITNs in the household requiring interventions that aim to increase the number of ITNs owned. Per RBM and WHO recommendations, programme activities should be directed towards increasing access to ITNs for all household members (ie. universal coverage).

The integrated campaigns targeted to children <5 years of age in Madagascar and Sierra Leone detected lower use by other age groups. Mathematical models have shown that targeting vulnerable groups does not necessarily result in adequate total population coverage to achieve a community effect (35-65% of the population sleeping under an ITN) [22]. Results presented here from these two countries suggest targeted campaigns may approach levels of population coverage needed to see a community effect (39.9% and 60.4%). More recently, many national malaria control programmes set a goal of universal coverage, but a standard definition and measurement tool has yet to be determined. Some definitions used for programmatic purposes include: two to three ITNs per household, one ITN for every two people, or one for every sleeping space in a household in an at-risk community. One measure, the proportion of all persons at risk sleeping under an ITN the previous night, was presented in this analysis.

The pattern of ITN use across age groups follow similar trends as published elsewhere [14,15]. Children 5-15 years were most likely to live in a household with an ITN hanging and yet not be sleeping under one. One possible explanation is that, consistent with IEC/BCC messages targeted to at-risk communities, the available ITNs are being used for younger siblings and/or pregnant women/mothers [23]. Another explanation may be the sleeping patterns and functional organization of the household [23,24]. Distribution of LLINs through schools may be a means to reach this population of older children [15]. On a positive note, many of the households had an ITN hanging, even if the child was not under it, which may provide some benefit due to repellent and insecticidal properties. Adults > 48 years were least likely to live in a household with an ITN and current approaches to ITN distribution may need to be modified (e.g. house-to-house methods) to more effectively reach this group and achieve universal coverage.

This analysis provides a useful platform to illustrate a common error made when interpreting M&E results. Using the standard RBM indicators, it may be tempting to consider the deficit in ITN use as the difference between the proportions of household ownership and individual use; however, this would be incorrect because they are calculated using two different denominators. For example, in Niger 65.1% of households owned an ITN and 55.5% of children < 5 years of age slept under an ITN. One may mistakenly conclude the 9.6% difference accounts for the deficit in use. Instead, using this framework one would make the more fitting conclusion that 22% of children < 5 years of age live in households owning an ITN but did not sleep under an ITN (16.4% in households with an ITN hanging and 5.6% not hanging). By classifying individuals into mutually exclusive groups using a common denominator, one is able to appropriately explore the underlying differences between ownership and use.

Likewise, one should use caution when drawing conclusions about the percentage of ITN use from ownership percentages. For example, only 60% of households owning an ITN does not imply a maximum of 60% of children under 5 are sleeping under an ITN. The use/ownership relationship depends on the distribution of children among households owning or not owning nets. Since a household with young children likely has a woman of reproductive age and may be more likely to have other young children as well, it is possible for usage to exceed ownership for certain age groups especially if children share the same sleeping spaces.

Also of note, the definition of a household may greatly affect ownership and reported use and should be considered carefully when comparing national surveys. Countries using definitions that include extended families or polygamous marriages may have a high probability of owning 'at least one ITN' due to the increased number of chances of obtaining a net through free distributions with each woman of reproductive age. However, they also have more household members and as a result each individual may be less likely to use a net because there are not enough nets available for everyone in the household. In contrast, if a household is defined as a mother and her children, then ownership levels may appear lower, but among households owning nets, an individual may be more likely to use it. These subtle differences in the definition of a household highlight a few of the programmatic considerations when interpreting the core indicators of household ITN ownership and individual use and challenge the move toward universal coverage.

The method presented here has important programmatic implications. First, similar to the common RBM indicators, this approach uses simple proportions to evaluate ITN use and does not require performing a logistic regression or other more sophisticated statistical analyses. Second, this approach can be performed using the data currently collected in the common national household surveys such as the MIS and DHS, without the need for additional questions. Lastly, results from this type of analysis can be used to guide programmatic strategies in terms of targeting deficits in ITN use. Population-based surveys can be analysed with this tool and help malaria control programmes select strategies that will help improve ITN use.

There are some potential limitations to the results presented in this paper. First, the survey data used in this analysis were not originally powered for subgroup analysis, resulting in some estimates having wide confidence intervals. Second, the timing of the surveys six to nine months after the campaigns may have introduced information/recall bias if respondents had difficulty remembering events from the campaign, misclassified the net type, or over-reported net use on the basis of social desirability. Third, households unavailable for an interview after multiple attempts or who refused to participate may introduce a non-response bias. Fourth, this paper did not look at the proportion of sleeping spaces covered, which could impact conclusions drawn about both adequate ownership and use. Lastly, although the current analysis looked at all members of the household, future studies could perform analyses accounting for household size and number of ITNs per household or per person, number of sleeping spaces covered; or investigate reasons for non-use among the specific categories described here [16].

## Conclusions

The core RBM bed net indicators, household ITN ownership and ITN use by vulnerable groups, play an important role in monitoring ITN distribution programmes. The framework described here supplements these primary indicators and provides an appropriate approach for investigating deficits in ITN use. Using data already available for the primary indicators, one can easily classify individuals into categories of ITN use and non-use with a common denominator (all individuals) and directly determine the distribution of persons among three types of non-use: not owning, not hanging, or not sleeping under an ITN. The framework proposed here uses information commonly collected from national household surveys (DHS, MIS), and as a result it can be readily incorporated into existing ITN monitoring and evaluation strategies to provide information critical to developing tailored activities aimed at improving ITN use.

## Competing interests

The authors declare that they have no competing interests.

## Authors' contributions

JVE contributed to the study design, participated in the acquisition and interpretation of data, performed the statistical analysis, and helped draft the manuscript. JT contributed to the design of the study, participated in the acquisition and interpretation of data, and helped draft the manuscript. MAK and AW contributed to the acquisition and interpretation of data, and helped review the manuscript. MA, ME, AH, and LS participated in the concept of the study and coordination, and provided critical review of the manuscript. All authors read and approved the final manuscript.

## Disclaimer

The findings and conclusions in this report are those of the authors and do not necessarily represent the views of the Centers for Disease Control and Prevention
